# Editorial: Computer vision in plant phenotyping and agriculture

**DOI:** 10.3389/frai.2023.1187301

**Published:** 2023-05-16

**Authors:** Ian Stavness, Valerio Giuffrida, Hanno Scharr

**Affiliations:** ^1^Department of Computer Science, University of Saskatchewan, Saskatoon, SK, Canada; ^2^School of Computing, Napier University, Edinburgh, United Kingdom; ^3^Institute for Advanced Simulation, IAS-8: Data Analytics and Machine Learning, Forschungszentrum Jülich, Jülich, Germany

**Keywords:** computer vision, plant phenotyping, deep learning, plant disease, datasets

## 1. Introduction

Plant phenotyping is the process of identifying a plant's structural and functional characteristics. Plant phenotyping is used by plant scientists to uncover mechanisms of plant physiology, e.g., to characterize how plants respond to biotic and abiotic stress. Phenotyping is also used by plant breeders to evaluate cultivars in a plant population for beneficial characteristics in order to inform the selection of progeny to move forward within a multi-year breeding process. In an attempt to reduce the time and cost required to phenotype large plant populations, image-based phenotyping has become popular over the past 10 years. Extracting phenotypic information from images of plants and crops presents a number of challenging real-world computer vision problems, such as analyzing images with highly self-similar repeating patterns and analyzing densely packed and occluded plant organs.

This Research Topic is associated with the 7th Computer Vision in Plant Phenotyping and Agriculture (CVPPA) workshop, which was held at the International Conference on Computer Vision (ICCV) on 11 October 2021. During the workshop, 18 full-length papers and 14 extended abstracts were presented. This Research Topic includes three papers that are extended versions of abstracts presented at the workshop. The Research Topic also includes 11 new articles that fall under the general scope of CVPPA but were not previously presented at the workshop.

The papers in this Research Topic explore a number of high-priority challenges in image-based phenotyping, including curating new datasets, developing few- and zero-shot analysis approaches that do not require extensive labeled datasets, handling occlusion in plant images, and visualizing and selecting appropriate models for plant phenotyping tasks. The collection of papers largely focuses on three areas: (1) general plant phenotyping tasks, such as plant species classification; (2) detection and classification of plant disease symptoms; and (3) detection of other plant pests including insects and weeds. Overall, this collection of high-quality papers has accomplished the goals of the Research Topic, which demonstrated the state-of-the-art research in image-based plant phenotyping, identified key unsolved problems, and introduced computer scientists to the field of plant phenotyping.

## 2. Papers

### 2.1. Plant phenotyping

A number of papers within this Research Topic investigated novel deep-learning and computer-vision techniques to tackle general plant phenotyping tasks and related technical challenges associated with collecting crop images. Fujiwara et al. reported a comparison if different approaches to estimate plant height from UAV images of outdoor maize fields. Liu K.-H. et al. proposed an efficient convolutional neural network (CNN) architecture for plant species classification from hyperspectral images. Zhang et al. combined field images and genotypic information for a population of sorghum cultivars toward elucidating genotype-by-phenotype interactions. With an image dataset of isolated Chrysanthemum flowers, Wang et al. investigated cultivar classification in a plant population with large morphological variations. Moving down in scale to images of individual seeds, Fonseca de Oliveira et al. reported on phenotyping of peanut seed quality. The final two papers reported novel deep learning approaches to plant phenotyping. Mostafa et al. proposed a new metric, the SSIM cut curve, for model selection in plant species classification. Kierdorf et al. used deep generative adversarial networks to reveal the likely plant organs that are hidden behind leaves in images of grapevines.

### 2.2. Disease detection

Many papers in this Research Topic explored approaches for detecting and recognizing disease symptoms from images of plants and plant organs. This matches a trend of increased interest in plant pathology in image-based plant phenotyping research and highlights the importance of biotic and abiotic stress phenotyping in modern crop breeding and farming operations. Papers in the collection have proposed new deep learning approaches to detect diseases in images of strawberries (Liao et al.), grapes (Suo et al.), maize (Qian et al.), rubber trees (Zeng et al.), and citrus trees (Yang et al.). Bruno et al. investigated adaptive minimal ensembling to achieve state-of-the-art performance on the well-studied PlantVillage leaf disease dataset. Finally, Egusquiza et al. proposed a metric learning approach to extract features from a small number of sample images. They demonstrated that the learned features have better discriminative and clustering properties as compared to a traditional supervised learning approach using a novel challenging leaf disease dataset.

### 2.3. Pest detection

A few papers in this Research Topic analyzed plant images to identify and count crop pests, including insects and weeds. Liu B. et al. proposed a new dataset of images of a wide range of forestry pests. Dai et al. also introduced a new pest image dataset but specialized for the Citrus psyllid pest, which is associated with the huanglongbing disease that is affecting citrus production worldwide. The authors reported a novel CNN approach to detect the tiny Citrus psyllid insects from citrus leaf images. Finally, Sapkota et al. evaluated the accuracy of transferring CNN models trained for detecting weeds in cotton crops to similar environments, but with soybean and maize crops. The adaptation and generalization of image-based plant phenotyping approaches to novel domains, such as different crop species or different environmental conditions, remains important challenges for the field.

## 3. Conclusion

To conclude, this Research Topic on Computer Vision in Plant Phenotyping and Agriculture has assembled a collection of papers that showcase a range of computer vision approaches and application domains. The authors would like to thank all the authors for their contributions to the Research Topic, and look forward to future research activity through the CVPPA workshop series.

## Author contributions

IS wrote the first draft of the manuscript. All authors contributed to manuscript revision, read, and approved the submitted version.

